# Intratumoral Immunotherapy—The Future of Treating Merkel Cell Carcinoma?

**DOI:** 10.3390/cancers18193226

**Published:** 2026-10-07

**Authors:** Nayonika Pande, Justin C. Moser, Sanjay Chandrasekaran, Andrew S. Brohl, Rajan P. Kulkarni, Vincent T. Ma

**Affiliations:** 1 University of Texas at Austin, Austin, TX 78712, USA; np6588@eid.utexas.edu; 2 HonorHealth Research Institute, Scottsdale, AZ 85258, USA; jmoser@honorhealth.com; 3 Arizona State John Shufeldt School of Medicine and Medical Engineering, Phoenix, AZ 85004, USA; 4 Department of Internal Medicine, University of Arizona College of Medicine, Phoenix, AZ 85004, USA; 5 Department of Internal Medicine, University of Texas Southwestern, Dallas, TX 75390, USA; sanjay.chandrasekaran@utsouthwestern.edu; 6 Moffitt Cancer Center, Tampa, FL 33612, USA; andrew.brohl@moffitt.org; 7 Department of Dermatology, Oregon Health and Sciences University, Portland, OR 97239, USA; kulkarnr@ohsu.edu; 8 Department of Medicine, Division of Hematology, Medical Oncology, and Palliative Care, University of Wisconsin-Madison, Madison, WI 53792, USA; 9 Department of Dermatology, University of Wisconsin-Madison, Madison, WI 53792, USA; 10 University of Wisconsin Carbone Cancer Center, 600 Highland Ave, K4/550, Madison, WI 53792, USA

**Keywords:** Merkel cell carcinoma, intratumoral therapy, abscopal response, immunotherapy, RECIST v1.1, itRECIST

## Abstract

Merkel cell carcinoma (MCC) represents a rare but highly aggressive neuroendocrine skin cancer with limited systemic therapy options for patients who fail or are ineligible for immune checkpoint inhibitors (ICIs). Intratumoral (IT) immunotherapy has emerged as a promising strategy, leveraging the accessibility of tumor lesions and their inherent capacity to trigger immune responses, known as abscopal effects. This review synthesizes the current evidence on IT approaches in MCC, including Toll-like receptor agonists, cytokines, and oncolytic viruses, and highlights their distinct mechanisms of action, while emphasizing the critical need for standardized radiographic response assessment of injected and non-injected lesions. Inconsistent documentation of abscopal responses has hindered quantification of IT therapy’s systemic potential, particularly in a disease where one-third of patients present with metastases and distant relapse is common. We outline MCC-specific rationale for routine lesion-level tracking, and recommendations for IT trial designs and objectives. Rigorous assessments of abscopal data will clarify IT’s role as both rescue therapy and an ICI partner, potentially transforming outcomes in this refractory MCC.

## Simple Summary

Merkel cell carcinoma is a rare but aggressive skin cancer that can be difficult to treat when standard immunotherapy does not work or cannot be safely given. Intratumoral immunotherapy, in which treatment is injected directly into a tumor, may stimulate the immune system not only at the injected site but also against tumors elsewhere in the body. In this review, we summarize the different types of intratumoral treatments studied in Merkel cell carcinoma, their potential benefits and limitations, and evidence that they may produce responses in tumors that were not directly injected. We also highlight the need for clinical trials to consistently measure responses in both injected and non-injected tumors. More standardized evaluation could help determine which intratumoral treatments provide meaningful systemic benefit and clarify how these therapies may be used alone or together with existing immunotherapies.

## 1. Introduction

Merkel cell carcinoma (MCC) is a rare and highly aggressive neuroendocrine cancer that is associated with high mortality. Its incidence in the United States remains low at approximately 0.8 cases per 100,000 individuals annually, compared with other cutaneous malignancies, and it carries a poorer prognosis. The five-year survival is about 50% overall, plummeting to under 15% in stage IV disease [1]. The established risk factors include advanced age, fair skin, cumulative ultraviolet (UV) radiation exposure, and chronic T-cell immunosuppression.

The carcinogenesis of MCC follows two distinct molecular pathways. Approximately 80% of cases are driven by the clonal integration of Merkel cell polyomavirus (MCPyV) into the host genome, leading to expression of viral oncoproteins, particularly large T and small T antigens, which are essential for tumor initiation and maintenance. The remaining 20% of cases are MCPyV-negative and characterized by a high tumor mutation burden resulting from chronic UV-induced DNA damage [2]. Despite these divergent origins, both subtypes demonstrate strong immunogenicity, making them highly susceptible to immune checkpoint inhibitor (ICI) therapy. The presence of tumor-infiltrating lymphocytes and MCPyV-specific CD8^+^ cytotoxic T cells has been consistently correlated with improved clinical outcomes, highlighting the central role of host immune surveillance in MCC pathophysiology [3].

## 2. Advancements in Systemic Immunotherapy for Merkel Cell Carcinoma

Historically, the only available systemic treatment of advanced or metastatic MCC was cytotoxic chemotherapy. Although objective responses could be achieved, durable disease control was uncommon. The introduction of ICIs has transformed the standard of care for unresectable and metastatic MCC. ICIs are particularly effective in MCC because these tumors are highly immunogenic yet adept at evading immune surveillance. MCC can suppress antitumor responses through multiple mechanisms, including the expression of immune checkpoint molecules such as PD-L1 on tumor cells and PD-1 on tumor-infiltrating lymphocytes [4]. MCC is associated with either the MCPyV or a high tumor mutation burden, either of which result in tumors harboring antigens that may make tumor cells recognizable to the immune system once inhibitory pathways are blocked. By releasing the immune “brakes”, ICIs revive exhausted T cells and restore their ability to recognize and eliminate tumor cells, leading to meaningful and durable clinical responses in many patients [5].

The ICIs such as avelumab, pembrolizumab, and retifanlimab that target the PD-1/PD-L1 pathway have shown objective response rates (ORRs) of approximately 33% in patients whose cancer resisted chemotherapy and around 56% in treatment-naïve advanced or metastatic MCC patients [6,7,8]. An alternative systemic option that can be used is dual ICI therapy. A meta-analysis of ipilimumab plus nivolumab showed a significantly higher pooled ORR than either anti-PD(L)1 monotherapy, when considering all anti-PD-1 and anti-PD-L1 agents (81.0% vs. 49.6%, *p =* 0.0001), as well as monotherapy when restricted to anti-PD-1 agents (81.0% vs. 57.0%, *p =* 0.0043) [9].

## 3. Rationale for Intratumoral Therapy: Why Intratumoral Treatment?

Despite the remarkable success of ICIs in MCC, up to half of patients either fail to achieve durable benefit or are inappropriate candidates for these treatments [6,7,8]. Some individuals cannot receive ICIs because of contraindications such as pre-existing autoimmune disorders or solid organ transplants and the need for chronic immunosuppression. Intratumoral immunotherapy may help to fill the clinical need in patients that are ineligible for systemic immunotherapy. For example, the ARTACUS trial demonstrated compelling antitumor activity of intratumoral RP1 for treatment of cutaneous malignancies in solid organ transplant recipients (SOTRs), a population for whom ICIs are generally not feasible [10]. Among patients who are eligible for and receive ICIs, a substantial proportion eventually experience disease progression due to primary or acquired resistance [6,8]. Within this context, IT or intralesional therapies have emerged as a rational, increasingly attractive strategy for the management of advanced MCC. IT immunotherapy represents a distinct treatment approach, in which the immunomodulatory agents are delivered directly into tumor lesions to locally modulate the tumor microenvironment and potentially induce systemic, abscopal antitumor effects, with relatively less systemic toxicities. Such properties position IT therapy as both a potential alternative for patients who are ineligible for ICIs and a promising partner in combination treatments designed to deepen and prolong responses. Because IT approaches may have discordant activity at injected and distant non-injected sites, there is a particular need to report tumor responses in greater detail, distinguishing outcomes in injected versus abscopal lesions using standardized criteria such as Response Evaluation Criteria in Solid Tumors (RECIST) v1.1 or intratumoral RECIST (itRECIST) [11]. Such precise reporting is essential to clarify the true systemic potential of IT strategies and to define their role alongside established systemic therapies in MCC.

## 4. Investigated Intratumoral Treatments in MCC

**Figure 1:**
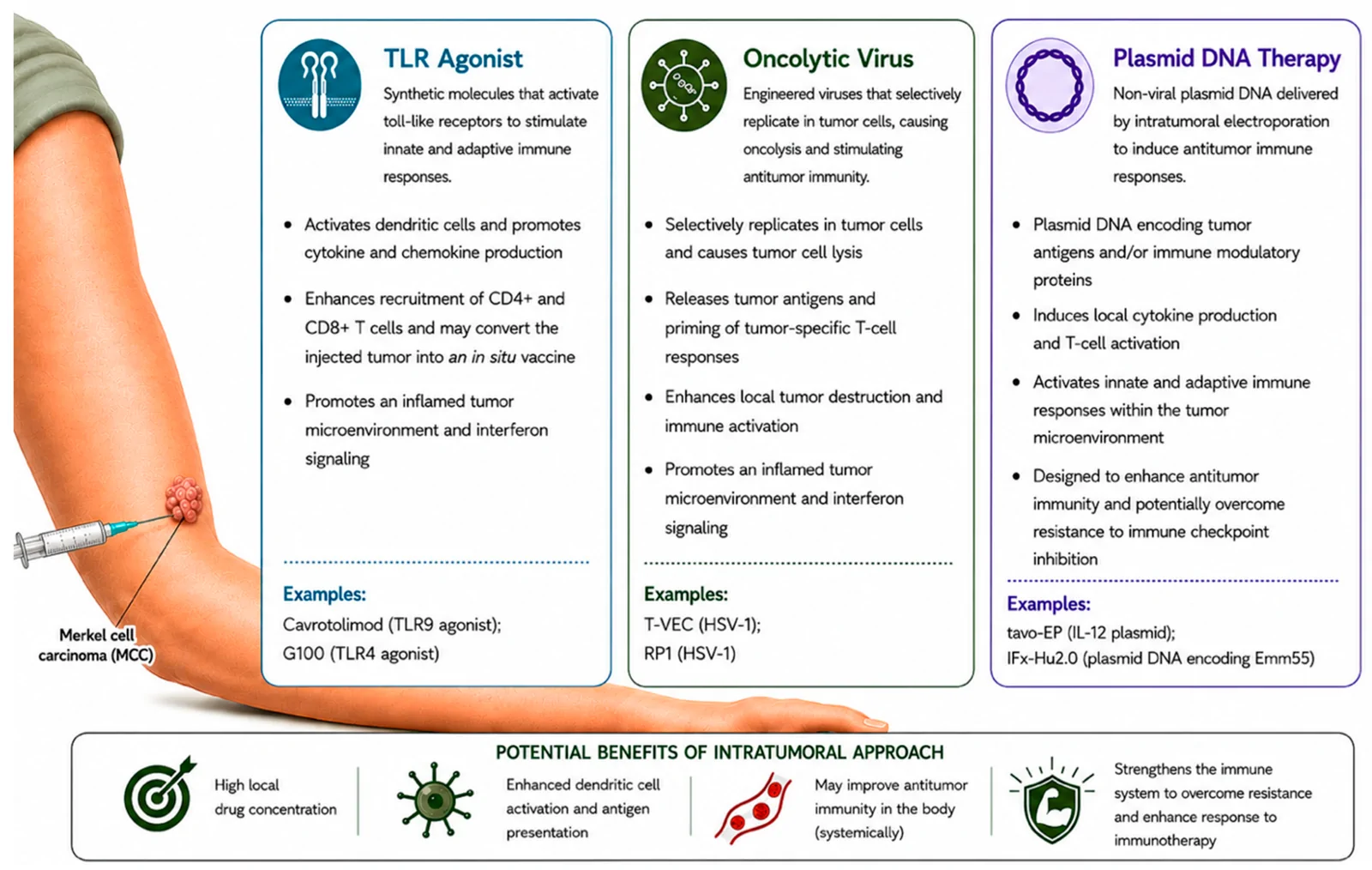
Intratumoral Agents Under Evaluation in MCC. Abbreviations: TLR, Toll-like receptor; HSV-1, herpes simplex virus type 1; IL, interleukin. This original figure was created in part using OpenAI, including AI-generated graphical assets. All content was reviewed and edited by the authors for scientific accuracy.

Multiple classes of intratumoral agents have been evaluated in MCC, including Toll-like receptor (TLR) agonists, cytokine-based approaches, and oncolytic viruses or vaccine-like platforms (Table 1 and Figure 1).

### 4.1. Intratumoral TLR Agonist

Cavrotolimod is a TLR9 agonist oligonucleotide formulated as a spherical nucleic acid (SNA^TM^) designed to enhance cellular uptake and stimulate both innate and adaptive antitumor immunity. After intratumoral injection, cavrotolimod activates plasmacytoid dendritic cells and other TLR9-expressing immune cells, leading to the production of type I interferons and pro-inflammatory cytokines/chemokines such as CXCL10 and IL-12p40, activation of T and NK cells into the tumor microenvironment, and systemic immune activation that can drive the regression of both injected and non-injected lesions, including in MCC [12]. In a Phase Ib/II trial of intratumoral cavrotolimod alone and in combination with anti-PD-1 (pembrolizumab or cemiplimab) in patients with advanced skin cancers, including a cohort of heavily pretreated, often PD-L1-refractory MCC, the regimen produced 1 complete response (CR) and one partial response (PR) among 18 MCC patients (11% ORR), with regression observed in both injected and non-injected tumors. Cavrotolimod demonstrated a manageable safety profile, with adverse events predominantly mild and brief (e.g., local injection-site reactions, flu-like symptoms, fatigue). Grade ≥ 3 treatment-related toxicities were uncommon, and no dose-limiting systemic cytokine-storm-type syndromes were reported, supporting repeated intratumoral administration in vulnerable populations [13].

G100 is an intratumoral formulation of glucopyranosyl lipid A (GLA), a synthetic TLR4 agonist delivered in a stable oil-in-water emulsion. After injection into tumors, G100 activates TLR4 on dendritic cells and other myeloid cells, driving local production of pro-inflammatory cytokines and chemokines, maturation of antigen-presenting cells, and recruitment of CD4^+^ and CD8^+^ T cells into the tumor microenvironment. This effectively converts the injected tumor deposit into an in situ vaccine that can prime systemic antitumor immunity and potentially induce abscopal effects in non-injected sites. In a pilot Phase I trial of intratumoral G100 in MCC, treatment was administered in both neoadjuvant and metastatic settings. The study required 1 lesion to remain non-injected throughout the study to monitor for abscopal effects. Among patients with stage IV MCC (*n* = 7), 2 patients achieved durable PRs (29% ORR) lasting over 33 months; 1 of the 3 patients with locoregional MCC treated with G100 in a neoadjuvant setting achieved a pathologic CR (33% ORR) and successfully completed surgery and adjuvant radiotherapy. G100 was generally well-tolerated in MCC patients, with adverse events predominantly limited to mild-to-moderate injection-site reactions (e.g., local erythema, swelling, pain) that were usually transient. Grade ≥ 3 toxicity was rare and consisted of localized skin reactions without systemic TLR4-mediated cytokine-release syndrome or treatment-related serious adverse events [3].

### 4.2. Intratumoral Cytokine-Based Therapies

Cytokine-based intratumoral strategies seek to harness the potent immunostimulatory properties of cytokines while reducing the systemic toxicities that have limited their traditional use. Interleukin-12 (IL-12), a key driver of T-helper 1-polarized immunity and cytotoxic T-cell activation, has been a central focus. To restrict IL-12 activity to the tumor site, an approach employing intratumoral injection of IL-12 plasmid DNA followed by in vivo electroporation (tavo-EP) has been developed. This technique achieves sustained local IL-12 expression with minimal systemic spread. These immune cells can then recognize and destroy tumor cells at the injected site and may also migrate to distant, noninjected lesions, supporting systemic antitumor immunity and potential abscopal effects. Tavo-EP has been evaluated in virus-associated skin cancers, including MCC. In a pilot study, patients with locoregional MCC (*n* = 3) who were candidates for definitive therapy (surgery ± radiotherapy) and metastatic MCC (*n* = 12) were treated. In patients with metastatic MCC, tavo-EP produced an ORR of approximately one quarter (3 of 12 patients), and regression of non-injected lesions (3 of 10 patients) was observed in a notable subset, supporting the induction of a systemic antitumor immune response. tavo-EP was generally well-tolerated in MCC, with adverse events largely limited to low-grade local injection and electroporation-related reactions (pain, erythema, edema). Exploratory biomarker analyses showed enhanced intratumoral T-cell infiltration and inflammatory gene signatures in many cases, supporting the mechanism of in situ vaccination and providing a framework to link lesion-level biology with abscopal responses [14].

### 4.3. Intratumoral Oncolytic Viruses and Vaccines

Talimogene laherparepvec (T-VEC) is an oncolytic herpes simplex virus approved for melanoma, has been used off-label and in small series for MCC, including in patients whose disease had progressed on PD-1/PD-L1 inhibitors. T-VEC is an attenuated herpes simplex virus type (HSV-1) oncolytic virus engineered to preferentially replicate in tumor cells and express GM-CSF. After intratumoral injection, T-VEC infects and lyses malignant cells, releasing tumor antigens in an environment enriched for GM-CSF-driven dendritic-cell recruitment and activation, which promotes priming of tumor-specific T cells capable of mediating regression of injected and non-injected MCC lesions [15]. In one report of 4 consecutive patients with regionally advanced, injectable MCC treated with intratumoral TVEC monotherapy, all 4 achieved durable complete responses, with progression-free survival beyond 16 months and ongoing overall survival beyond 18 months in most cases [16]. A report of 2 cases on the utilization of combination therapy T-VEC + PD-1/PD-L1 inhibitor in MCC patients with anti-PD-1 refractory disease. One patient had a CR, and another patient had a near-CR.17 Most recently, a single-institution retrospective study evaluating advanced nonmelanoma skin cancers (NMSC) reported outcomes for 10 MCC patients. The cohort achieved a 60% ORR, comprising 5 CRs and 1 PR. The remaining 4 patients experienced progressive disease (PD). Of the 6 responders, 1 received IT T-VEC in conjunction pembrolizumab, while 5 received T-VEC monotherapy [17]. In an interim analysis of a Phase II study in refractory cutaneous lymphoma and advanced or refractory NMSC, patients received IT T-VEC for up to 12 weeks, with nivolumab added only for non-responders. T-VEC monotherapy showed limited overall efficacy across tumor types, though activity was noted in patients without visceral metastases. Two PRs (20% ORR) occurred in MCC patients without visceral disease [18]. In another Phase II trial tumor response was assessed by modified World Health Organization criteria in 1 largest non-injected, non-irradiated metastatic lesion from solid tumor following IT T-VEC with (*n* = 10) or without RT (*n* = 9); 2 patients with MCC were treated in each study arm. Responses in the largest untreated lesion were uncommon and were observed in 10–11% of patients per study arm (1 responder per arm); notably, both responders were patients with MCC, though no objective responses were reported [19]. Taken together, these reports suggest that T-VEC-based therapy can induce clinical responses, including in combination with checkpoint blockade, supporting the idea that viral oncolysis coupled with local expression of immunostimulatory factors may help restore sensitivity in otherwise refractory disease.

RP1 (vusolimogene oderparepvec) is an oncolytic HSV-1-based immunotherapy engineered to selectively infect and lyse tumor cells while stimulating antitumor immunity; it also carries GM-CSF and a fusogenic glycoprotein to enhance local tumor destruction and immune activation. Preclinical and translational data from the same program suggest that RP1 promotes an inflamed tumor microenvironment and interferon signaling, which may help to sensitize tumors to PD-1 blockade [20]. In the phase I/II IGNYTE study, intratumoral RP1 given with nivolumab produced encouraging activity including complete and partial responses in anti-PD-1 naïve (4 CRs; 67% ORR) and anti-PD-1 refractory (5 CRs, 3 PRs; 30% ORR) MCC patients, supporting further clinical development of this regimen [21]. In the Phase 1b/II ARTACUS trial, which enrolled SOTRs with advanced cutaneous squamous cell carcinoma (cSCC; *n* = 24) and MCC (*n* = 3), single-agent RP1 demonstrated compelling antitumor activity, achieving a 35% ORR and a 22% CR rate among evaluable patients, RP1 monotherapy was well-tolerated, with the most common adverse events of fatigue (33%), chills (26%), and pyrexia (26%) [10].

### 4.4. Other Therapies

IFx-Hu2.0 is an intratumoral plasmid DNA immunotherapy that encodes the bacterial protein Emm55. After direct injection into tumor lesions, it is intended to activate innate and adaptive immune responses within the tumor microenvironment and help to overcome resistance to ICI. In the Phase Ib study of patients with advanced MCC or cSCC previously treated with ICI, IFx-Hu2.0 was followed by post-protocol ICI rechallenge in several MCC patients, and 5 of 9 evaluable MCC patients (56%) achieved an objective response to ICI rechallenge despite prior resistance. These findings suggest a potential immune-priming or re-sensitization effect in MCC. IFx-Hu2.0 was reported to be safe and feasible with weekly dosing for up to 3 weeks and was generally well-tolerated [22]. A Phase II/III randomized, double-blind, placebo-controlled study in ICI naïve advanced or metastatic MCC is currently ongoing.

MIW815 (ADU-S100) is a synthetic cyclic dinucleotide agonist of STING (Stimulator of Interferon Genes) pathway. It induces type I interferon signaling, activates dendritic cells, enhances tumor antigen cross-presentation, and promotes tumor-specific CD8^+^ T-cell responses. In the first-in-human, Phase I study, 47 patients with advanced or metastatic solid tumor or lymphomas were treated including 3 MCC patients. Of the 3 MCC patients treated, 1 had a confirmed PR providing early clinical evidence of antitumor activity with STING agonism. Although overall single-agent efficacy was limited, systemic immune activation was demonstrated by increased inflammatory cytokines and T-cell clonal expansion [23]. Treatment was generally well-tolerated with the treatment-related most common adverse events reported as pyrexia (17%), chills, injection-site pain (15%), and headache (13%).

PH-762 is a self-delivering RNAi therapeutic designed to reduce PD-1 expression locally within the tumor microenvironment, thereby releasing a key immune checkpoint brake on tumor-infiltrating T cells. Preclinical data show that intratumoral PH-762 decreases PD-1 mRNA and protein in tumor-infiltrating lymphocytes, increases CD4^+^ and CD8^+^ T-cell infiltration, and promotes IFN-γ and CXCL10 signaling, supporting an in situ immune-activating effect [24].

Recently, intratumoral administration of the anti-PD-1 antibody cemiplimab has been investigated in patients with early-stage cSCC in the ongoing Phase III CLEAR study, which is evaluating intratumoral cemiplimab as a potential alternative to primary surgery. Although a similar approach has not yet been evaluated in MCC, the biological rationale is compelling. MCC is a highly immunogenic malignancy in which systemic PD-1/PD-L1 blockade has demonstrated substantial clinical activity. As such, investigation of intratumoral PD-1 blockade in the neoadjuvant setting may represent a promising strategy to enhance local and systemic antitumor immunity while potentially reducing disease burden before definitive treatment. Studies modeled after the CLEAR design could help define the feasibility, efficacy, and immunologic effects of this approach in patients with localized MCC [25].

As of October 2026, several ongoing and actively recruiting clinical trials are evaluating novel therapeutic strategies for MCC, including approaches in both the frontline and treatment-refractory settings (Table 2). Collectively, these investigations support the concept that intratumoral immunotherapy can effectively remodel the tumor microenvironment, overcome local immune resistance, and, in some patients, trigger systemic antitumor immunity. These IT strategies may serve both as a critical option for patients who cannot receive standard systemic immunotherapy and as an enhancing component of combination regimens designed to optimize outcomes in Merkel cell carcinoma.

## 5. Abscopal Effect

A goal of IT immunotherapy is not only to treat the injected lesion, but to initiate an antitumor immune response that could become systemic. Local administration of IT agents such as TLR agonists, cytokines, or oncolytic viruses promotes tumor cell death and the release of tumor-associated antigens. These antigens are captured by activated antigen-presenting cells, leading to the priming and expansion of tumor-specific T cells. These effector cells can then circulate and attack distant, untreated tumor deposits [26]. Clinically, this phenomenon is described as an abscopal effect, in which non-injected lesions regress following local IT treatment of one or a few select, accessible, and clinically relevant tumor lesions [27]. Although biologically plausible, the abscopal effect remains controversial as it is uncommon, inconsistently observed, and difficult to distinguish from other concurrent treatment effects [28].

MCC provides a compelling framework for the evaluation of abscopal effects of IT immunotherapy. MCC often presents with accessible cutaneous lesions as potential portals for systemic immune activation. A substantial proportion of MCC patients ultimately develop regionally advanced or metastatic disease, and approximately one-third present with nodal or distant involvement at diagnosis [29]. Even after apparently successful locoregional management, distant relapse remains common and is frequently fatal. In this context, IT therapies that could reliably induce abscopal responses, shrinkage, or clearance of non-injected lesions would offer a way to leverage accessible cutaneous disease to achieve systemic control. Evidence of abscopal activity has begun to emerge from early-phase MCC studies. In the tavo-EP trial, for example, non-injected lesions regressed in a meaningful subset of patients, including cases with PRs driven by the shrinkage of multiple distant metastases. Correlative studies demonstrated increased recruitment of MCPyV-specific T cells in non-injected lesions, providing a mechanistic link between local treatment and its distant effect. Similarly, the immune remodeling observed with G100, and the responses reported with T-VEC in checkpoint-refractory disease, are consistent with the activation of systemic antitumor immunity rather than a purely local cytotoxic effect. As such, careful documentation and reporting of abscopal effects in MCC are critical, as these observations may guide patient selection, inform trial design, response data collection, analyses, reporting, and help refine the role of intratumoral treatments.

An additional area of interest is the integration of IT treatment with radiotherapy, which is commonly used in the treatment of patients with MCC. Radiotherapy may enhance antigen release from tumor cells, while IT therapies can amplify the resulting immune response within the tumor microenvironment and potentially at distant disease sites. Consequently, the combination may increase the likelihood of an abscopal effect compared with either modality alone. This approach is currently being evaluated in several clinical studies, including trials combining radiotherapy with T-VEC and GI-101.

### Predictive Biomarkers and Strategies to Enhance the Abscopal Effect

At present, no biomarker has been prospectively validated to predict which patients with MCC will develop an abscopal response after intratumoral therapy. However, several biologically plausible candidates merit prospective study. Baseline and on-treatment features of an immune-inflamed tumor microenvironment, including CD8^+^ T-cell infiltration, expansion of tumor-reactive T-cell clones, interferon signaling, and MCPyV-specific T-cell responses, may reflect the capacity for local immune activation to become systemic. In the G100 and tavo-EP studies, treatment-induced immune infiltration and immune activation accompanied responses in injected and, in some patients, non-injected lesions [3,14]. Serial circulating tumor DNA and MCPyV oncoprotein antibody titers are validated markers of disease burden and recurrence risk in MCC, but they should currently be viewed as pharmacodynamic or surveillance tools rather than established predictors of abscopal response [30,31]. Future intratumoral trials should prospectively integrate paired biopsies of injected and non-injected lesions, blood-based biomarkers, and longitudinal immune profiling to determine whether these features predict systemic benefit.

Strategies to increase the probability of an abscopal response may include combining intratumoral therapy with immune checkpoint blockade to sustain and expand tumor-reactive T cells, or with radiotherapy to increase antigen release and inflammatory signaling. Radiotherapy may also influence tertiary lymphoid structures (TLSs) in a context-dependent, bidirectional manner. Immunogenic cell death, cGAS-STING/type I interferon signaling, vascular remodeling, and lymphocyte recruitment may support TLS-associated antitumor immunity, whereas high-dose or large-volume irradiation and nodal exposure may damage lymphocytes, tumor-draining lymph nodes, stromal scaffolds, or high endothelial venules and thereby impair TLS formation or maturation [32]. These effects remain hypothesis-generating and warrant prospective evaluation in MCC, particularly when radiotherapy is combined with intratumoral immunotherapy. These combinations may also confound attribution of responses in non-injected lesions. Accordingly, combination trials should incorporate designs that can isolate the contribution of the intratumoral agent, prespecified lesion-level endpoints, and biomarker sampling before and after treatment.

## 6. Importance of Systematic Response Assessment of Non-Injected Tumor Lesions

One of the key objectives in evaluating IT therapies for MCC and more broadly should be to distinguish between local efficacy at injected sites and systemic, or abscopal, effects observed in non-injected lesions. In most modern IT trial design, abscopal response data are reported but can be challenging due to the inherent limitations of trial response assessments [27].

Traditional response criteria, RECIST v1.1, do not differentiate between injected and non-injected lesions, limiting their ability to capture the distinct local and systemic components of IT efficacy. The itRECIST framework addresses this need by classifying all measurable lesions at baseline as either target-injected (T-I) or target-non-injected (T-NI) [11]. As itRECIST provides a standardized method to evaluate both aspects independently, it thereby improves the interpretability and comparability of results across trials involving intratumoral therapies. In addition, itRECIST permits the use of ultrasound for measurement of superficial and subcutaneous lesions, which are common in MCC and may be difficult to accurately size using conventional cross-sectional imaging. However, this approach is associated with greater operator dependence and the potential for inter-site variability in measurements which may affect reproducibility across multicenter studies.

To assess systemic activity, the sum of diameters (SOD) of T-NI lesions should be tracked over time and compared with both baseline and nadir measurements, following the conventional RECIST v1.1 methodology. Responses in these lesions may arise from local immune activation, concurrent anticancer systemic therapy, or their combination. In contrast, responses in injected lesions are assessed iteratively. Because the set of injected lesions can change during treatment due to factors such as regression or accessibility, their SOD at each time point is compared to the immediately preceding assessment rather than the baseline measurement.

For these reasons, future studies of IT therapy in MCC should prospectively define injected and non-injected target lesions and report RECIST v1.1 outcomes separately for each category. Detailed abscopal response data, including response rates, time to response, and duration of response in non-injected disease.

Additionally, for comprehensive visualization and reporting, itRECIST recommends the use of dual waterfall plots to illustrate the maximum percentage change in SOD for both injected and non-injected target lesions separately. This dual approach ensures that local therapeutic effects do not obscure potential systemic immune responses and allows outcomes to be compared consistently across clinical studies.

Circulating tumor DNA (ctDNA) is a promising molecular biomarker in MCC for monitoring treatment response and disease recurrence. A serial ctDNA monitoring can provide a sensitive, minimally invasive method for assessing molecular residual disease, identifying patients at increased risk of recurrence, and evaluating the efficacy of systemic or local therapies. This test has the potential to complement conventional imaging and clinical assessment in the management of MCC and has been recommended by the 2025 NCCN guidelines.

## 7. Recommendations for Future IT Clinical Trials

- IT trials in MCC should prospectively define target lesions as injected versus non-injected at baseline and report RECIST-based outcomes separately for each group, ideally incorporating itRECIST to better distinguish local treatment effects from systemic responses. Cutaneous lesions should be documented with standardized photography, complemented by imaging for visceral and nodal disease sites.
- Trial objectives should be clearly defined, including abscopal response rate (proportion of non-injected target lesions achieving RECIST v1.1 PR/CR), time to onset, and duration of response in non-injected lesions. Whether injected lesions will be included in tumor response assessment or not should be clearly defined in the efficacy endpoints. Such standardized detail will not only strengthen the clinical interpretability of abscopal effects but also enhance mechanistic understanding.
- Future studies could incorporate paired biopsies of injected and non-injected lesions together with exploratory immune-monitoring endpoints, such as T-cell receptor sequencing and MCPyV-specific immune assays, to better characterize treatment-induced immune responses. Integrating these biomarkers with assessments of ctDNA and immune cell profiling may help define the relationship between local immune modulation and systemic antitumor activity. Prospective incorporation of these assessments as exploratory endpoints, with correlation to clinical outcomes, could improve understanding of whether local tumor changes induced by ITT are associated with systemic immune activation and antitumor effects.
- In trials where IT drugs are combined with other drugs:
  ○ Use a design that isolates the IT contribution rather than evaluating only the combination’s activity.
  ○ An approval-enabling combination study should include a control or comparator arm.
  ○ Enroll a homogenous patient population, use stratification for disease stage (III vs. IV), only cutaneous/superficial vs. visceral disease, high- vs. low-volume disease, etc., as appropriate.
  ○ Select the primary endpoint capable of demonstrating a meaningful clinical benefit from investigational IT therapy. For objective assessment, include a blinded independent central review. The endpoints to evaluate the mechanism of action can be secondary.

## 8. Discussion

IT immunotherapy is emerging as a promising strategy in the management of MCC, based on its success in melanoma, where IT therapy has already received regulatory approval. IT therapies have the ability to induce localized immune activation and potentially generate systemic antitumor responses, known as the abscopal effect. This strategy is particularly crucial in MCC, a highly immunogenic disease with limited durable responses to current systemic therapies.

The regression of non-injected tumor lesions has been observed in a subset of patients in IT clinical studies, suggesting that localized immune modulation can translate into systemic therapeutic benefit. However, the magnitude of these responses in non-injected lesions remains variable, emphasizing the need for modified trial designs and the standardization of response evaluation. The incorporation of modified response criteria, itRECIST, is an important step toward addressing the unique challenges associated with IT therapy assessment. Standardized use of itRECIST may improve regulatory evaluation, cross-trial comparability, and future meta-analyses.

The clinical development of intratumoral therapy differs substantially across cutaneous malignancies. In melanoma, talimogene laherparepvec has randomized phase III data and regulatory approval, with lesion-level analyses showing greater regression in injected lesions than in uninjected non-visceral or visceral lesions, providing a benchmark for quantifying local and systemic effects [33]. Neoadjuvant talimogene laherparepvec has also been evaluated prospectively before surgery in resectable melanoma [34]. In cutaneous squamous cell carcinoma, intralesional development has increasingly extended into earlier-stage primary disease, including the phase III CLEAR cSCC study comparing intralesional cemiplimab with surgery [25,35]. By contrast, the MCC literature remains dominated by small phase I/II cohorts and case series. The strong immunogenicity of MCC and frequent accessibility of cutaneous or superficial nodal lesions provide a compelling rationale, but cross-tumor response rates should not be compared directly because patient populations, disease burden, injected sites, agents, and response criteria differ.

Within MCC, there are insufficient data to determine whether injecting a primary tumor produces a different systemic effect than injecting a cutaneous, subcutaneous, or nodal metastasis. The G100 pilot illustrates both settings: one of three patients treated in the locoregional/neoadjuvant cohort achieved a pathologic complete response, whereas two of seven patients with metastatic disease achieved durable partial responses [3]. These small numbers do not establish a site-specific efficacy difference. Conceptually, treatment of an intact primary tumor before surgery may provide an opportunity to prime immunity while the tumor and its draining lymphatic network remain in place, whereas injection of skin or superficial nodal metastases is typically used in established regional or systemic disease, where the objective may include both local control and induction of distant responses. Future trials should record whether injected lesions are primary tumors, in-transit or skin metastases, nodal metastases, or visceral lesions and analyze outcomes by injection site.

Combination strategies are also likely to be central to future intratumoral therapy development in MCC. Systemic checkpoint blockade is the most developed partner, as illustrated by RP1 plus nivolumab, reports of talimogene laherparepvec with PD-1/PD-L1 blockade, and IFx-Hu2.0 followed by checkpoint inhibitor rechallenge [21,22,36]. Radiotherapy is another rational partner because it can increase tumor antigen release and inflammatory signaling, while intratumoral therapy may amplify local immune activation and facilitate distant responses [19]. Conventional cytotoxic chemotherapy is less established as a partner; chemotherapy can reduce tumor burden, but responses in MCC are generally short-lived, and there is currently no prospective evidence that adding chemotherapy to intratumoral immunotherapy improves outcomes. Neoadjuvant use is particularly attractive because it may reduce tumor burden before definitive local therapy, generate systemic immunity while tumor antigen remains present, and enable paired tissue analyses. The G100 pilot provides early MCC-specific proof of concept, while neoadjuvant talimogene laherparepvec in melanoma demonstrates the feasibility of this strategy in another cutaneous malignancy [3,34]. Prospective MCC-specific studies are needed to define the optimal partner, sequencing, and disease setting.

Safety profiles observed in IT trials have generally been favorable compared with systemic therapies. Most adverse events are localized, including injection-site pain, inflammation, and peripheral edema. Systemic symptoms are flu-like illness or fatigue and are mild and self-limited. Severe grade toxicities are uncommon but may occur with certain delivery platforms, such as electroporation, or when IT therapies are combined with checkpoint inhibitors. In combination regimens, overlapping toxicities require careful monitoring to distinguish local inflammatory reactions from systemic immune-related adverse events. The overall tolerability of IT therapy makes it an option for patients who cannot tolerate intensive systemic regimens (e.g., transplant recipients, patients with severe autoimmune conditions, and older or frail patients). In addition, many IT therapies can be administered in the outpatient setting.

Despite these encouraging findings, several practical and biological challenges persist. A significant proportion of IT clinical trials have focused on cutaneous and subcutaneous lesions because of the relative ease of direct injection. In contrast, the treatment of deep visceral metastatic lesions may require image-guided procedures and carries procedural risks, including bleeding, infection, pain, injury to adjacent structures, organ perforation, pneumothorax, and anesthesia-related complications. The image-guided delivery with interventional radiology support often requires multidisciplinary collaboration highlighting the importance of the multidisciplinary tumor board to carefully select appropriate candidates, coordinate cross-specialty logistics, and manage potential toxicities. Ensuring consistent and complete drug delivery to deep-seated lesions over multiple treatment cycles remains a critical barrier that may limit broader applicability. The priority should be to expand IT strategies to deep-seated visceral lesions, explore combination regimens with checkpoint inhibitors, and develop biomarker-driven approaches to identify patients who may benefit.

The recent FDA review of the RP1 melanoma program also underscores evidentiary challenges relevant to intratumoral therapy development. Although RP1 plus nivolumab produced durable responses in anti-PD-1-refractory melanoma and activity has been reported in MCC, the FDA’s 2026 review identified uncertainty related to the single-arm design, heterogeneity of the treated population, reliability of historical controls, response-assessment methodology, and the ability to determine the contribution of RP1 relative to nivolumab [37]. The sponsor has emphasized the durability of responses and submitted additional analyses intended to address contribution of effect and injected versus non-injected lesion responses [38]. These differing interpretations do not negate the observed clinical activity, but they illustrate why future approval-enabling intratumoral studies should prospectively define injected and non-injected lesions, document systemic responses rigorously, and, when feasible, incorporate randomized or otherwise interpretable comparator strategies capable of establishing the contribution of the intratumoral agent.

Overall, IT therapy is a promising and evolving therapeutic approach in MCC. Its ability to combine localized treatment with the potential for abscopal activity while maintaining a favorable safety profile makes it an important treatment strategy. Continued refinement of clinical trial designs and deeper insights into mechanisms of action are crucial to maximize its clinical impact.

## Figures and Tables

**Table 1:** Intratumoral Agents and Trials in MCC.

Agent	Mechanism/Class	Clinical Trial(s)	Patient Population (*n*)	Overall RECIST v1.1 Response	Abscopal Signals (Non-Injected Lesions)
Cavrotolimod	TLR9 agonist	NCT03684785 (Phase Ib/II)	ICI-refractory solid tumors including MCC (MCC-enriched cohort; *n* = 18)	1 CR 1 PR	Abscopal effect in both non-injected and regional distant tumors
G-100	TLR4 agonist	NCT02035657 (Phase I/II pilot)	Locoregional/neoadjuvant (*n* = 3) and Metastatic MCC (*n* = 7)	1 pCR 2 PR	Non-injected lesion regressions reported in responders
tavo-EP	Cytokine (IL-12 plasmid DNA + electroporation)	NCT01440816 (Phase II pilot)	Locoregional (*n* = 3) and Metastatic MCC (*n* = 15)	3 PR	≥30% Regression of non-injected distant lesions in 3/10 patients
T-VEC (Talimogene laherparepvec)	Oncolytic virus (HSV-1 + GM-CSF)	Off-label case series/small cohorts; single-institution retrospective study; NCT02978625 (Phase II, T-VEC followed by T-VEC + nivolumab for non-responders); NCT02819843 (Phase II, T-VEC +/− radiotherapy)	ICI-refractory MCC case reports/series (*n* = 6); advanced nonmelanoma skin cancer cohort including MCC (*n* = 10); NCT02978625 (*n* = 10 MCC); NCT02819843 (*n* = 4 MCC)	Case reports/series: 4 CR with T-VEC alone and 1 CR/1 near-CR with T-VEC + PD-1/PD-L1 blockade; retrospective cohort: 5 CR/1 PR among 10 MCC patients; NCT02978625: 2 PR; NCT02819843: no objective responses	Non-injected lesion responses were not consistently reported. In NCT02819843, one patient in each arm had a response in the largest untreated metastatic lesion.
RP1 (Vusolimogene oderparepvec)	Oncolytic virus (HSV-1)	NCT03767348 (Phase I/II; IGNYTE) RP1 + nivolumab	Anti-PD-1 naïve (*n* = 6 MCC) Anti-PD-1 failed (*n* = 27 MCC)	Anti-PD-1 naïve (4 CR) Anti-PD-1 failed (5 CR, 3 PR)	Not specified
RP1 (Vusolimogene oderparepvec)	Oncolytic virus (HSV-1)	NCT04349436 (Phase I/II; ARTACUS) RP1 monotherapy	MCC in solid organ transplant recipients (*n* = 3)	Not specified for MCC	Not specified
MIW815 (ADU-S100)	STING-Agonist	NCT02675439 (Phase I; Dose escalation)	Advanced metastatic solid tumors and lymphoma (*n* = 3 MCC)	1 PR	Not specified
IFx-Hu2.0	Plasmid DNA encoding for an immunogenic bacterial protein, Emm55	NCT04160065 (Phase Ib)	ICI refractory advanced/metastatic MCC (*n* = 13)	None	Not specified

CR: complete response; PR: partial response; pCR: pathological complete response; RT: radiotherapy.

**Table 2:** Active and/or Recruiting Studies for MCC.

Investigational Agent(s)	NCT Number	Agent Type	Phase	Patient Population (Stage/Type)	Recruitment Status
IFx-Hu2.0 + pembrolizumab	NCT06947928	Intratumoral immunostimulant	Phase II/III	Advanced/metastatic MCC, ICI-naïve	Recruiting
IFx-Hu2.0 + pembrolizumab	NCT06940440	Intratumoral immunostimulant	Phase I	Advanced/metastatic MCC, ICI- naïve	Recruiting
VET3-TGI ± pembrolizumab	NCT06444815	Novel immunotherapy	Phase I	Advanced solid tumors (includes MCC)	Recruiting
MEM-288 ± nivolumab	NCT05076760	Oncolytic virus	Phase I	Advanced solid tumors (includes MCC)	Recruiting
PH-762	NCT06014086	ICI	Phase I	Advanced Stage IV MCC, ICI refractory	Recruiting
MQ710 (MQ719) ± pembrolizumab	NCT05859074	Immune-modulating agent	Phase I	Solid tumors (includes MCC)	Recruiting
GI-101 ± pembrolizumab/lenvatinib/RT	NCT04977453	IL-2/IL-15 fusion protein	Phase I/II	Advanced solid tumors (includes MCC)	Recruiting
T-VEC + nivolumab	NCT02978625	Oncolytic virus	Phase II	Advanced/metastatic NMSC (includes MCC)	Active

## Data Availability

No new data were created or analyzed in this study. Data sharing is not applicable to this article.
